# Physical Inactivity and Incidence of Obesity among South Australian Adults

**DOI:** 10.1371/journal.pone.0112693

**Published:** 2014-11-10

**Authors:** Alicia M. Montgomerie, Catherine R. Chittleborough, Anne W. Taylor

**Affiliations:** 1 Discipline of Medicine, School of Medicine, The University of Adelaide, Adelaide, Australia; 2 Discipline of Public Health, School of Population Health, The University of Adelaide, Adelaide, Australia; Alberta Provincial Laboratory for Public Health/University of Alberta, Canada

## Abstract

The aim of this paper is to examine the association of physical inactivity with incidence of obesity in the South Australian adult population. Two representative data sources were used – the South Australian Monitoring and Surveillance System (SAMSS), a monthly surveillance system, and the North West Adelaide Health Study (NWAHS), a biomedical cohort study. There were 75.3% (n = 12873) SAMSS participants and 72.8% (n = 1521) of NWAHS participants that were not obese at baseline. The cumulative incidence of obesity for SAMSS participants from the previous year to the current year was 2.7%. The cumulative incidence of obesity for NWAHS participants between baseline and stage 3 was 14.4%. Physical inactivity was associated with incident obesity (RR 1.48, 95% CI 1.14–1.90 [SAMSS] and RR 1.41, 95% CI 1.03–1.93 [NWAHS]). This association remained, but was attenuated after adjustment for chronic conditions, risk factors and socio-demographic factors. However, physical activity should be continued to be encouraged in the population for its known additional health benefits.

## Introduction

Obesity is a major public health concern with the prevalence rates doubling and even tripling in many countries in the last three decades [1]. The prevalence of obesity in Australian adults was 28.3% in 2011–2012 [2]. Obesity has been linked to an increased risk of premature death and conditions that have an adverse effect on quality of life [3]. These conditions include diabetes, cardiovascular diseases, musculoskeletal disorders, stroke, some cancers, a number of respiratory and gastrointestinal diseases, osteoarthritis, and some mental health conditions [4]–[6]. Health risks associated with obesity impose a considerable economic burden on individuals, families and communities, with the total annual direct cost of overweight and obesity in Australia estimated to be $58.2 billion in 2008 [7]. In recognition of this, the Australian Government endorsed obesity as a national health priority area in 2008 [8].

There is evidence that the increase in obesity prevalence is associated with a decrease in physical activity [9]–[11]. The overall prevalence of Australian adults reporting low or sedentary levels of physical activity was 66.9% in 2011–12 [12]. In 2009 it was reported that 27% of physically inactive Australian adults were obese, compared to only 16% of people undertaking a high level of physical activity [13]. The Australian Diabetes, Obesity and Lifestyle Study (AusDiab) reported a positive association between obesity and lower physical activity [14]. Previous cross-sectional studies have also reported an inverse relationship between physical activity and body mass index (BMI) [15]–[20]. A recent study among US adults reported that obesity incidence was significantly higher among adults who did not participate in leisure time activity [21]. However, these studies are cross-sectional and causality cannot be determined from the observed association. From these studies, it is not clear whether physical inactivity causes obesity or whether physical inactivity is a consequence of obesity. A longitudinal study of adults in Copenhagen reported that there was no evidence that physical inactivity promotes the development of obesity [22]. Evidence of the association of physical inactivity and the development of obesity from other prospective observational population studies that measure physical activity at baseline, are few and give inconsistent results [23]–[26].

There are currently no nationally representative longitudinal studies on obesity and its association with physical inactivity, and more specifically there are no studies on the incidence of obesity and physical inactivity within the South Australian population. Prevalence data are important for conveying the magnitude of the obesity problem, but incidence conveys information about the rate of its development. By examining the association of physical inactivity with incident cases of obesity, we can be clearer about the causal nature of physical inactivity in the development of obesity.

The aim of this paper is to examine the association of physical inactivity with incidence of obesity in the South Australian adult population. Providing evidence for the link between physical inactivity and incident obesity may strengthen the argument for improving physical activity among Australians, in an effort to prevent people becoming obese. We used two data sources to examine this association: one cross-sectional which relies on retrospective, self-reported data to establish obesity incidence, and the other a longitudinal cohort study following the same people prospectively over time.

## Methods

### Samples

This paper used two existing representative South Australian data sources of adults aged 18 years and over. The first data source is the South Australian Monitoring and Surveillance System (SAMSS); a monthly chronic disease and risk factor telephone survey of randomly selected people established in July 2002 [27]. All households in South Australia with a telephone number listed in the Electronic White Pages are eligible for selection in the sample. Within each household, the person who had a birthday last is selected for interview. There is no replacement for non-contactable persons. Data are collected by a contracted agency using Computer Assisted Telephone Interviewing (CATI) and interviews are conducted in English. Detailed SAMSS methodology has been published elsewhere [28]. We used a weighted SAMSS sample from data collected in the period January 2008 to December 2011 from participants aged 18 years and over (n = 22,535). The response rate of SAMSS for this period was between 60% and 65% each month.

The second data source is the North West Adelaide Health Study (NWAHS), a representative population cohort study of 4056 randomly selected adults aged 18 years and over recruited from the northern and western regions of Adelaide. This region represents approximately half of the metropolitan area and one-third of the population in South Australia [29]. Detailed methodology of the study has been previously reported [30]. All households in the north-western area of Adelaide with a telephone connected and the telephone number listed in the Electronic White Pages were eligible for selection. Within each household, the person who had their birthday last and was 18 years or older, was selected for interview and invited to attend the clinic. Baseline assessment (Stage 1) of the cohort occurred between December 1999 and July 2003 with 4056 adults participating in the clinical assessment, resulting in an overall response rate of 49.4%. The first follow-up (Stage 2) of the cohort occurred between May 2004 and February 2006 with survey data collected for 88% (n = 3574) and clinic data for 79% (n = 3206). The second follow-up (Stage 3) of the cohort occurred between June 2008 and August 2010, with survey data collected for 67% (n = 2710) and clinic data for 61% (n = 2487) of eligible participants. The same survey methodologies were used at all stages of the study. Self-reported demographics and health-related information was collected in the CATI survey and a self-completed questionnaire. Biomedical data were obtained at the clinic appointment.

### Obesity

SAMSS participants were asked, ‘What is your height without shoes?’, ‘What is your weight? (Undressed in the morning)’ and ‘How much did you weigh a year ago?’ Participants in the NWAHS cohort had their height measured without shoes using a wall mounted stadiometer in centimetres to the nearest 0.5 centimetre and weight measured on calibrated scales in kilograms to the nearest 0.1 kilogram. BMI was calculated as weight in kilograms divided by the square of height in metres. Current BMI for SAMSS participants was calculated from self-reported current weight and height, and BMI in the previous year was calculated from self-reported weight in the previous year and current self-reported height. BMI was categorised according to the World Health Organizationrganisation criteria [5]. Participants with a BMI greater than or equal to 30 kg/m^2^ were classified as obese [5].

### Physical inactivity

SAMSS participants were asked to recall physical activity behaviours in the past week on the time they spent undertaking walking, moderate and vigorous activity for sport, recreation or fitness from items from the validated Active Australia Questionnaire [AAQ] [31]–[34]. Moderate exercise was defined as activity undertaken for fitness, recreation or sport that causes a moderate increase in heart rate or breathing, while vigorous exercise was defined as causing a large increase in a person's heart rate or breathing. The National Physical Activity Guidelines for Australians recommends accumulating 30 minutes of moderate or greater intensity physical activity on most days of the week, translated as 150 minutes per week [35]. Sufficient Physical Activity (SPA) can thus be defined as the completion of 150 minutes of walking, moderate and vigorous activity (with vigorous multiplied by two to account for its greater intensity) in the past week [31]. Participants not achieving SPA were classified as physically inactive. In the NWAHS at baseline, physical activity was assessed from the self-completed questionnaire using items from the Australian Bureau of Statistics (ABS) National Health Survey that required participants to recall physical activity behaviours in the past two weeks [36]. Questions focused on the typical frequency, intensity and duration of walking for sport, recreation or fitness, moderate and vigorous exercise, where moderate exercise was any exercise which caused a moderate increase in heart rate or breathing and vigorous exercise was any exercise which caused a large increase in heart rate or breathing. From these components, an exercise score was calculated by frequency of exercise sessions multiplied by the average time per session multiplied by intensity, where walking has an intensity score of 3.5, with 5.0 for moderate exercise and 7.5 for vigorous exercise. Scores were classified as sedentary (below 100), low (between 100 and below 1600), moderate (scores from 1600 to 3200, or greater than 3200 and less than two hours of vigorous exercise) and high (over 3200 and more than two hours of vigorous exercise), as determined by the ABS [33]. Participants with a score of sedentary or low were classified as physically inactive.

### Covariates

In both SAMSS and NWAHS, the socio-demographic characteristics included were age (in years), sex, country of birth (born in Australia; not born in Australia), highest educational attainment (Bachelor degree or higher; trade, apprenticeship, certificate or diploma; secondary education or less), marital status (married or living with partner; separated, divorced, widowed, never married), gross annual household income (more than $60,000; less than or equal to $60,000), work status (employed; not employed), and socioeconomic status as measured by the Socio Economic Indexes for Areas (SEIFA) Index of Relative Disadvantage [37]. SEIFA was dichotomised into most advantaged (middle, high or highest quintile) and most disadvantaged (lowest or low quintile). For NWAHS analyses, region (western; northern) was also included.

For NWAHS participants, diabetes was defined as having fasting plasma glucose of at least 7.0 mmol/L, or reporting having been told by a doctor that they have diabetes in the self-completed questionnaire [38], Asthma was based on three pre- and three post-salbutamol measurements, and defined as at least a 12% increase in FEV1 (forced expiratory volume in one second) from pre-Ventolin to post-Ventolin if the absolute difference in FEV1 was greater than 200 ml, or an absolute change in FEV1 of greater than or equal to 400 ml [39], or if participants reported having been told by a doctor that they have asthma. COPD was defined as a post bronchodilator FEV1:FVC ratio of less than 70% [40]. For SAMSS participants self-reported doctor diagnosed health conditions included diabetes, arthritis, osteoporosis, asthma (diagnosed with asthma by a doctor and experienced symptoms and/or treatment in the last 12 months [41]), and chronic obstructive pulmonary disease (COPD, chronic bronchitis or emphysema). For both NWAHS and SAMSS participants cardiovascular disease (CVD) was defined as being told by a doctor that they had ever had a heart attack, a stroke or angina and current mental health condition was defined as a doctor diagnosis of anxiety, depression, a stress related problem or any other mental health problem in the last twelve months.

Risk factors included smoking (current smokers; non- or ex-smokers), alcohol consumption, high cholesterol, high blood pressure and self-reported general health status (NWAHS and SAMSS), as well as fruit, vegetable and fast food consumption and psychological distress (SAMSS only). Alcohol consumption was classified according to the National Health and Medical Research Council guidelines for the risks associated with consuming alcohol, where lifetime risk of harm from alcohol-related disease or injury was those participants who drank two or more standard drinks in one occasion and for alcohol related injury risk, those participants who drank four or more standard drinks in one occasion [42]. High cholesterol for NWAHS participants was determined from a fasting blood sample taken at the study clinic and defined as total blood cholesterol greater than or equal to 5.5 mmol/L [43]. SAMSS participants were classified as having high cholesterol if they reported having been told by a doctor that they had high cholesterol. High blood pressure for NWAHS was defined as systolic blood pressure greater than or equal to 140 mmHg and/or diastolic blood pressure greater than or equal to 90 mmHg [44]. SAMSS participants were classified as having high blood pressure if they reported having been told by a doctor that they had high blood pressure and/or if they were on antihypertensive treatment [45]. Self-reported general health status [46] asked participants to rate their general health as excellent, very good, good, fair or poor; dichotomised into excellent, very good or good, vs. fair or poor. Insufficient daily vegetable or fruit consumption was defined as having consumed less than five serves of vegetables, or less than two serves of fruit (SAMSS). High fast food consumption was classified as more than one serve of fast food per week (SAMSS). SAMSS participants were classified as having high or very high levels of psychological distress if they had a score of 22 or above on the Kessler 10 Psychological Distress Scale [47].

### Data analysis

**Figure 1:**
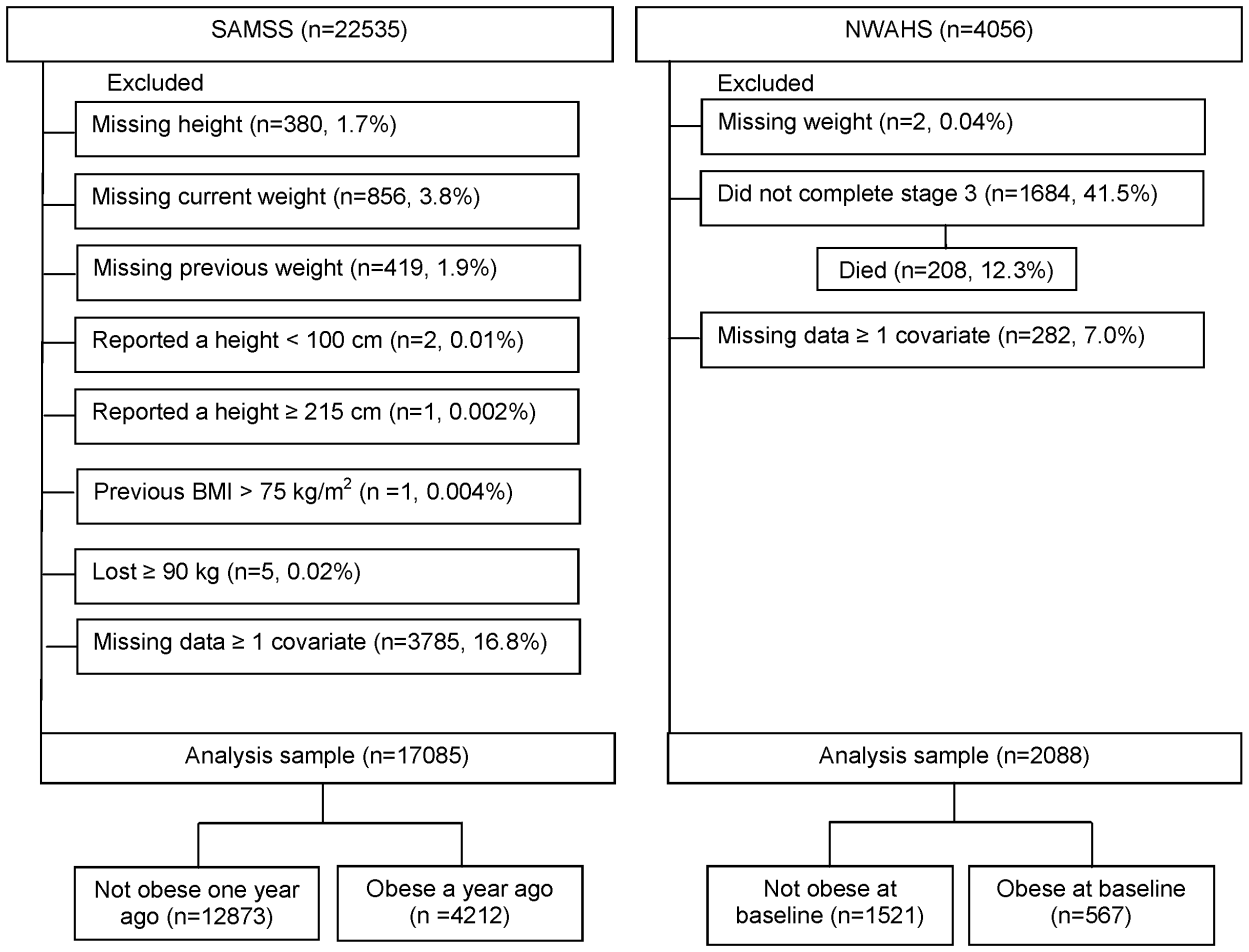
South Australian Monitoring and Surveillance System (SAMSS) and North West Adelaide Health Study (NWAHS) study sample.

SAMSS data were weighted by age, sex, area and probability of selection in the household to estimated resident population data so that the results were representative of the South Australian population. We excluded participants with missing data for height (n = 380), current (n = 856) or previous weight (n = 419), those who reported a height ≥215 cm (n = 1) or <100 cm (n = 2), those who had a previous high BMI (>75 kg/m2, n = 1), those who lost ≥90 kg (n = 5) and those missing data for at least one covariate (n = 2998). This yielded a final study size of n = 17,085 (Figure 1).

NWAHS data were weighted to the Australian Bureau of Statistics' Census [48] and Estimated Residential Population for South Australia [49], by region, age group, sex, and probability of selection in the household, to provide population representative estimates. We excluded participants who were missing data on weight (n = 2), those that did not complete stage 3 (n = 1684, of which n = 208 had died) and those missing data for at least one covariate (n = 282). This yielded a final study size of n = 2088 (Figure 1).

Data were analysed using Statistical Package for the Social Sciences (SPSS) version 19 for Windows (Chicago, IL) and STATA version 10.0 (College Station, TX). Cumulative incidence of obesity in SAMSS was calculated by dividing the number of participants who were currently obese (BMI ≥30 kg/m^2^) by the number of participants who were not obese (BMI <30 kg/m^2^) one year ago. Cumulative incidence of obesity in NWAHS was calculated by dividing the number of participants who became obese at Stage 3 by the number of participants who were not obese at baseline (Stage 1). Incidence of obesity was also reported per 1000 person years, taking into account the different follow-up time periods in SAMSS (12 months) and NWAHS (mean follow-up time 2898.9 days, SD [Standard Deviation] 402.29).

Generalised linear regression models using the log link were used to estimate the relative risk (RR) for the association of physical inactivity and incident obesity. Model 1 was adjusted for age and sex. Model 2a was adjusted for age, sex and chronic conditions (diabetes, asthma, COPD, cardiovascular disease, mental health) and model 2b was adjusted for age, sex and chronic conditions as in model 2a and also adding arthritis and osteoporosis from SAMSS. Model 3a was adjusted for age, sex, chronic conditions and risk factors (smoking, alcohol, SF-1, blood pressure and cholesterol) and model 3b was adjusted for age, sex, chronic conditions and risk factors as in model 3a and also adding vegetable consumption, fruit consumption, fast food consumption and psychological distress from SAMSS. Model 4a was adjusted for age, sex, chronic conditions, risk factors and demographics (SEIFA, country of birth, education, marital status, income and work status) and model 4b was adjusted for age, sex, chronic conditions, risk factors and demographics as in model 4a and region from NWAHS.

Ethics approval was granted by the Government of South Australia SA Health Human Research Ethics Committee (HREC/13/SAH/44).

## Results

The baseline characteristics of NWAHS and SAMSS study participants who were obese or not obese are described in Table 1. In the baseline SAMSS sample one year before the interview, 24.7% (n = 4212) were classified as obese (BMI ≥30 kg/m^2^), 35.6% (n = 6079) were classified as overweight (BMI 25.0–29.9 kg/m^2^), 37.4% (n = 6397) were classified as normal weight (BMI 18.50–24.99 kg/m^2^) and 2.3% (n = 397) were classified as underweight (BMI <18.50 kg/m^2^). At baseline, 27.2% (n = 567) of NWAHS participants were classified as obese (BMI ≥30 kg/m^2^), 38.3% (n = 799) were classified as overweight (BMI 25.0–29.9 kg/m^2^), 33.8% (n = 706) were classified as normal weight (BMI 18.50–24.99 kg/m^2^) and 0.8% (n = 16) were classified as underweight (BMI <18.50 kg/m2).

**Table 1:** Socio-demographic characteristics of participants who were obese and not obese, South Australian Monitoring and Surveillance System (SAMSS) and North West Adelaide Health Study (NWAHS).

	SAMSS			NWAHS		
	% or mean ± SD			% or mean ± SD		
	Overall (n = 17085)	Not obese one year ago (n = 12873)	Obese one year ago (n = 4212)	Overall (n = 2088)	Not obese at baseline (n = 1521)	Obese at baseline (n = 567)
Age (years)	48.4±17.06	47.9±17.71	49.6±14.81	45.5±15.78	44.6±16.22	47.8±14.28
Male (%)	50.6	51.1	49.1	49.2	50.6	45.6
Lowest and low quintile (IRSD) (%)	36.1	33.4	44.4	54.4	51.9	61.3
Born in Australia (%)	79.2	78.7	80.8	71.2	73.1	70.5
Secondary education or less (%)	47.7	46.5	51.4	42.1	41.1	44.9
Married (%)	71.3	70.4	73.9	69.6	68.4	72.8
Household income ≤$60,000 (%)	43.4	41.7	48.4	71.0	69.0	76.2
Employed (%)	66.1	66.5	64.7	62.6	64.8	56.8
Western region (%) (NWAHS only)				44.8	48.1	36.2

SD: Standard Deviation, IRSD: Index of Relative Disadvantage

Note: Socio-demographic characteristics are current for SAMSS participants and baseline for NWAHS participants. Obesity status is reported one year ago for SAMSS participants and measured at baseline for NWAHS participants.

There were 75.3% (n = 12873) of SAMSS participants who were not obese at baseline and 72.8% (n = 1521) of NWAHS participants who were not obese at baseline. The cumulative incidence of obesity for SAMSS participants from the previous year to the current year was 2.7% (Table 2). The cumulative incidence of obesity for NWAHS participants between baseline and Stage 3 was 14.4% (Table 2). Incidence of obesity was higher among those who were physically inactive (SAMSS 3.3%; NWAHS 16.3%) compared to those who were physically active (SAMSS 2.3%; NWAHS 11.5%) (Table 2). Weight gain among SAMSS participants who became obese (n = 353) was greater (mean 9.2 kg, SD 6.99) than among the participants who did not become obese (n = 12,520, mean 0.3 kg, SD 3.72). Weight gain among NWAHS participants who became obese (n = 219) was greater (11.7 kg, SD 8.55) than among the participants who did not become obese (n = 1302, 1.2 kg, SD 5.98).

**Table 2:** Obesity incidence overall, and by physical activity, South Australian Monitoring and Surveillance System (SAMSS) and North West Adelaide Health Study (NWAHS).

	n	events	Cumulative incidence (95% CI)	Incidence rate per 1000 person-years
SAMSS Obesity	12873	353	2.7 (2.5–3.0)	27.4
Physically active	7209	163	2.3 (1.9–2.6)	22.7
Physically inactive	5664	189	3.3 (2.9–3.8)	33.5
NWAHS Obesity	1521	219	14.4 (12.7–16.3)	18.1
Physically active	607	70	11.5 (9.2–14.3)	15.0
Physically inactive	914	149	16.3 (14.0–18.8)	20.2

CI: Confidence Interval.

Physical inactivity was associated with incident obesity (RR 1.48, 95% CI 1.14–1.90 [SAMSS] and RR 1.41, 95% CI 1.03–1.93 [NWAHS], Table 3). This association remained, but was attenuated, after adjustment for chronic conditions, risk factors and socio-demographic factors.

**Table 3:** Association of physical inactivity with incident obesity, South Australian Monitoring and Surveillance System (SAMSS) and North West Adelaide Health Study (NWAHS).

	SAMSS (n = 12873)		NWAHS (n = 1521)	
	RR (95% CI)	P-value	RR (95% CI)	P-value
Basic model (unadjusted)	1.48 (1.14–1.90)	0.003	1.41 (1.03–1.93)	0.031
Model 1^a^	1.51 (1.17–1.95)	0.002	1.39 (1.02–1.92)	0.039
Model 2a^b^	1.43 (1.10–1.85)	0.007	1.42 (1.03–1.95)	0.030
Model 2b^c^	1.36 (1.05–1.77)	0.022	-	-
Model 3a^d^	1.30 (1.00–1.69)	0.050	1.32 (0.96–1.81)	0.093
Model 3b^e^	1.24 (0.94–1.64)	0.125	-	-
Model 4a^f^	1.26 (0.97–1.64)	0.083	1.27 (0.15–1.76)	0.920
Model 4b^g^	-	-	1.26 (0.91–1.73)	0.164

RR: relative risk. CI: Confidence Interval.

a Adjusted for age and sex.

b Adjusted by age, sex and chronic conditions (diabetes, asthma, COPD, cardiovascular disease and mental health).

c Adjusted by age, sex and chronic conditions (diabetes, asthma, COPD, cardiovascular disease, mental health, arthritis [SAMSS only] and osteoporosis [SAMSS only]).

d Adjusted for age, sex, chronic conditions and risk factors (smoking, alcohol, SF-1, blood pressure and cholesterol).

e Adjusted for age, sex, chronic conditions and risk factors (smoking, alcohol, SF-1, blood pressure, cholesterol, vegetable consumption [SAMSS only], fruit consumption [SAMSS only], fast food consumption [SAMSS only] and psychological distress [SAMSS only].

f Adjusted for age, sex, chronic conditions, risk factors and sociodemographics (SEIFA, country of birth, education, marital status, income, work status).

g Adjusted for age, sex, chronic conditions, risk factors and sociodemographics (SEIFA, country of birth, education, marital status, income, work status and region [NWAHS only]).

## Discussion

In these two representative population studies of South Australia adults, we found that physical inactivity is associated with incident obesity but this association is attenuated when adjusted for confounders. Physical inactivity was associated with incident obesity using both data sources. The association was attenuated when the models were adjusted for risk factors and socio-demographic factors. The relative risks for the association of physical inactivity and incident obesity are similar in each model for both studies. Smaller P-values observed with the SAMSS data could be explained by the larger sample size. By including a retrospective question of weight in SAMSS, a surveillance system, we are able to continually monitor and identify those at high risk of becoming obese to target prevention efforts.

The attenuation in the model including fruit, vegetable and fast food consumption using SAMSS data is consistent with recent studies reporting that weight gain is explained more by increased energy intake than physical inactivity [50], [51]. Other studies have also demonstrated associations between physical inactivity and obesity but do not provide evidence that physical inactivity promotes the development of obesity [22], [52]–[54]. More recently, results from the Whitehall II study reported that there was no association between sitting time and incident obesity [55].

The difference in incidence rate per 1000 person years between SAMSS and NWAHS may be explained by the fact that NWAHS participants and their nominated general practitioner received a result letter after each clinic assessment. The letter highlighted any results outside the healthy range, and indicated that the desirable level for BMI should be between 18.50 and 24.99. This feedback on their health may have made participants more aware of their weight and prompted them to make changes in order to prevent them becoming obese. In the NWAHS there were more obese participants at baseline than SAMSS participants which may also explain the difference in the incidence rate. The cumulative incidence for SAMSS was consistent with a similar study in the US (4.0%) using the same retrospective weight question [21]. The cumulative incidence for NWAHS was consistent with another prospective cohort study (15.3%) with that had a median follow-up of 6.4 years [56].

A strength of this study is that it utilised two South Australian data sources of adults aged 18 years and over to examine the association of physical inactivity and incident obesity. Both studies have a large random sample, and participants are representative of the whole State (SAMSS) or a large region of South Australia. In addition, the NWAHS has measured height and weight and other biomedical measures at more than one time point.

A limitation of this study is that BMI for SAMSS participants was based on self-reported weight and height, and previous studies have shown that height is generally over-estimated and weight is under-estimated [57], [58]. SAMSS participants were also asked to recall weight in the past year, however self-reported past weight has been shown to be a reasonably reliable measure of past body weight [59]–[62]. The samples were restricted to participants with complete data on height, weight and covariates. There was also loss to follow-up for NWAHS participants, although a proportion (n = 208) of the attrition in this sample is mortality related. This attrition could cause selection bias if the relationship between physical inactivity and obesity is different between participants and non-participants [63] but we have no reason to suggest that this may be the case. We also made the assumption that current health behaviours and socio-demographic information of SAMSS participants accurately reflected their health behaviours and socio-demographic information in the previous year. The recall periods for physical activity behaviours differed between the two studies, with NWAHS participants asked about physical activity undertaken in the last two weeks, whereas SAMSS participants were asked about physical activity undertaken in the past week. Self-reported measures of physical activity are limited due to recall error, and social desirability; however the items have been shown to have acceptable reliability and validity for population level physical activity [32]–[34]. Physical activity in these two studies only includes exercise for sport, recreation or fitness, and therefore is not necessarily indicative of total physical activity; for example it excludes physical activity at work. The studies also do not take into account the time participants spent being sedentary. A recent study has found that time spent sitting for prolonged periods may not only be detrimental to people's health but may also counteract the benefits of regular moderate to vigorous physical activity [64]. The studies also lack data on participants' complete dietary intake, so only limited dietary factors could be included in our models.

## Conclusions

In conclusion, we found that physical inactivity is associated with incident obesity but this association is attenuated when adjusted for confounders. Further study is needed to take into account sedentary time, physical activity at work and dietary behaviours and incident obesity. Physical activity should, however, continue to be encouraged in the population for its known additional health benefits [65].
